# Automated Acetabular Defect Reconstruction and Analysis for Revision Total Hip Arthroplasty: A Computational Modeling Study

**DOI:** 10.1002/jor.26086

**Published:** 2025-05-02

**Authors:** Daniel Hopkins, Stuart A. Callary, L. Bogdan Solomon, Peter V. S. Lee, David C. Ackland

**Affiliations:** ^1^ Department of Biomedical Engineering University of Melbourne Parkville Victoria Australia; ^2^ Centre for Orthopaedic and Trauma Research University of Adelaide Adelaide South Australia Australia; ^3^ Department of Orthopaedics and Trauma Royal Adelaide Hospital Adelaide South Australia Australia

**Keywords:** artificial neural network, biomechanical model, hip surgery, statistical shape modeling

## Abstract

Revision total hip arthroplasty (rTHA) involving large acetabular defects is associated with high early failure rates, primarily due to cup loosening. Most acetabular defect classification systems used in surgical planning are based on planar radiographs and do not encapsulate three‐dimensional geometry and morphology of the acetabular defect. This study aimed to develop an automated computational modeling pipeline for rapid generation of three‐dimensional acetabular bone defect geometry. The framework employed artificial neural network segmentation of preoperative pelvic computed tomography (CT) images and statistical shape model generation for defect reconstruction in 60 rTHA patients. Regional acetabular absolute defect volumes (ADV), relative defect volumes (RDV) and defect depths (DD) were calculated and stratified within Paprosky classifications. Defect geometries from the automated modeling pipeline were validated against manually reconstructed models and were found to have a mean dice coefficient of 0.827 and a mean relative volume error of 16.4%. The mean ADV, RDV and DD of classification groups generally increased with defect severity. Except for superior RDV and ADV between 3A and 2A defects, and anterior RDV and DD between 3B and 3A defects, statistically significant differences in ADV, RDV or DD were only found between 3B and 2B‐2C defects (*p* < 0.05). Poor correlations observed between ADV, RDV, and DD within Paprosky classifications suggest that quantitative measures are not unique to each Paprosky grade. The automated modeling tools developed may be useful in surgical planning and computational modeling of rTHA.

## 1 Introduction

Acetabular defects caused by osteolysis, infection, wear, implant removal or previous surgery present a significant technical challenge in revision total hip arthroplasty (rTHA) [1]. While primary THA is regarded as a successful treatment option for osteoarthritis, with an expected implant survival rate of 96% at 10 years [2], rTHA survivorship has been reported as low as 72% at 10 years [3], primarily due to acetabular implant loosening [4] after reconstructions of large acetabular defects [5]. Several radiographic acetabular defect grading systems, including the Paprosky classification [6], are used to assist in clinical decision making for defect reconstructions [7]. However, since these classifications systems are based on assessment of planar radiographs, and do not represent three‐dimensional (3D) anatomy observed intraoperatively [8], they may adversely influence surgeon preparedness and ability to surgically manage defect reconstruction.

Variations in defect volume and shape within a given defect classification may be considerable, particularly in cases of large defects such as Paprosky grade 3B which exhibit high variability in bone loss [9]. Computational methods such as 3D geometric bone modeling and statistical shape modeling (SSM) can provide quantitative analysis of acetabular defects and lead to higher interobserver repeatability of classifications [10]; however, these methods rely on manual digital segmentation of computed tomography (CT) images which is time consuming and typically not practical in the clinical setting [11].

Fast and efficient strategies for accurate quantification of defect morphology from CT scans are not currently available [11], but have potential to assist in surgical planning and component selection for rTHA, especially as individualized reconstructions are gaining popularity [12]. While general acetabular anatomy associated with Paprosky classifications is well established, quantitative data including defect volume and depth, and their variability, remain poorly understood. The aims of this study were twofold. First, to develop a fully automated modeling pipeline to reconstruct acetabular defects from clinical CT images and validate it against manually derived reconstructions; and second, to use this framework to evaluate and compare defect volume and depth within and between Paprosky classification grades. We hypothesized that defect volume and depth within the Paprosky classifications would be associated with significant overlap between classification grades.

## 2 Materials and Methods

### 2.1 Patient Recruitment and Medical Imaging

One hundred and fifteen patients undergoing rTHA at the Royal Adelaide Hospital between June 2010 and January 2023 by one surgeon (LBS) were prospectively recruited for this study. All patients had a preoperative CT scan and were screened for inclusion using the following criteria: the presence of acetabular defects, a full view of both sides of the pelvis, sufficient slice thickness (1 mm or below) and sufficient ability to delineate the boundary between acetabular trabecular bone and surrounding tissue or fluid, including in the presence of metal artifact. This resulted in exclusion of 29 patients due to inadequate scan protocol (wrong field of view or inadequate slice thickness) and 26 patients due to excessive metal artifact (Supporting Information: Figure S1). The final CT data set comprised 60 patients (mean age 72.1 ± 10.0 years; 28 female, 32 male; mean weight 85.2 ± 20.5 kg) (Table 1). The number of previous acetabular revisions for all patients ranged from zero to a maximum of four (no previous revisions: 26 subjects, one or more previous revisions: 34 subjects). Slice thickness of CT images ranged from 0.5 mm to 1.0 mm with a median of 0.7 mm. Where available, CT image processing was employed using a metal artifact reduction algorithm (34 patients), and soft tissue reconstruction kernels (28 patients), since this allowed clearer differentiation between trabecular bone and the surrounding tissue. Images were manually segmented to reconstruct the hemipelvis of the affected acetabulum (Mimics, Materialise, Belgium). Preoperative CT scans of left rTHAs were flipped to the right side to standardize model orientation. All images were cropped to include only the pelvis and 10 slices above the iliac crest and 10 slices below the base of the ischium. In all cases preoperative acetabular defects were classified according to Paprosky by the operating surgeon as previously described [6]. Ethical approval for this study was provided by Central Adelaide Local Health Network (Ref: 16717).

**Table 1:** Summary of cohort demographics and preoperative clinical data.

	*N*	%
Age (years)		
50–59	8	13.3
60–69	13	21.7
70–79	24	40.0
80–89	14	23.3
90–99	1	1.7
Gender		
Female	28	46.7
Male	32	53.3
Paprosky classification		
2A	2	3.3
2B	14	23.3
2C	13	21.7
3A	14	23.3
3B	17	28.3
Previous revisions		
0	26	43.3
1	10	16.7
2	7	11.7
3	3	5.0
4	3	5.0
Index primary THA unknown	11	18.3
Reason for current revision		
Infection	12	20.0
Loosening	22	36.7
Osteolysis	10	16.7
Fracture	3	5.0
Recurrent dislocation	1	1.7
Dislocated liner	1	1.7
Implant fracture	1	1.7
Not reported	3	5.0
Unrevised to date	7	11.7
Failed cup material		
PE	24	40.0
Metal	35	58.3
Not reported	1	1.7
Cemented/uncemented		
Cemented	25	41.7
Uncemented	34	56.7
Not reported	1	1.7
Operation year		
2011	1	1.7
2012	1	1.7
2013	4	6.7
2014	3	5.0
2015	4	6.7
2016	2	3.3
2017	1	1.7
2018	2	3.3
2019	6	10.0
2020	8	13.3
2021	4	6.7
2022	9	15.0
2023	2	3.3
Unrevised to date	7	11.7

Abbreviations: PE, polyethylene; THA, total hip arthroplasty.

### 2.2 Acetabular Defect Reconstruction

**Figure 1:**
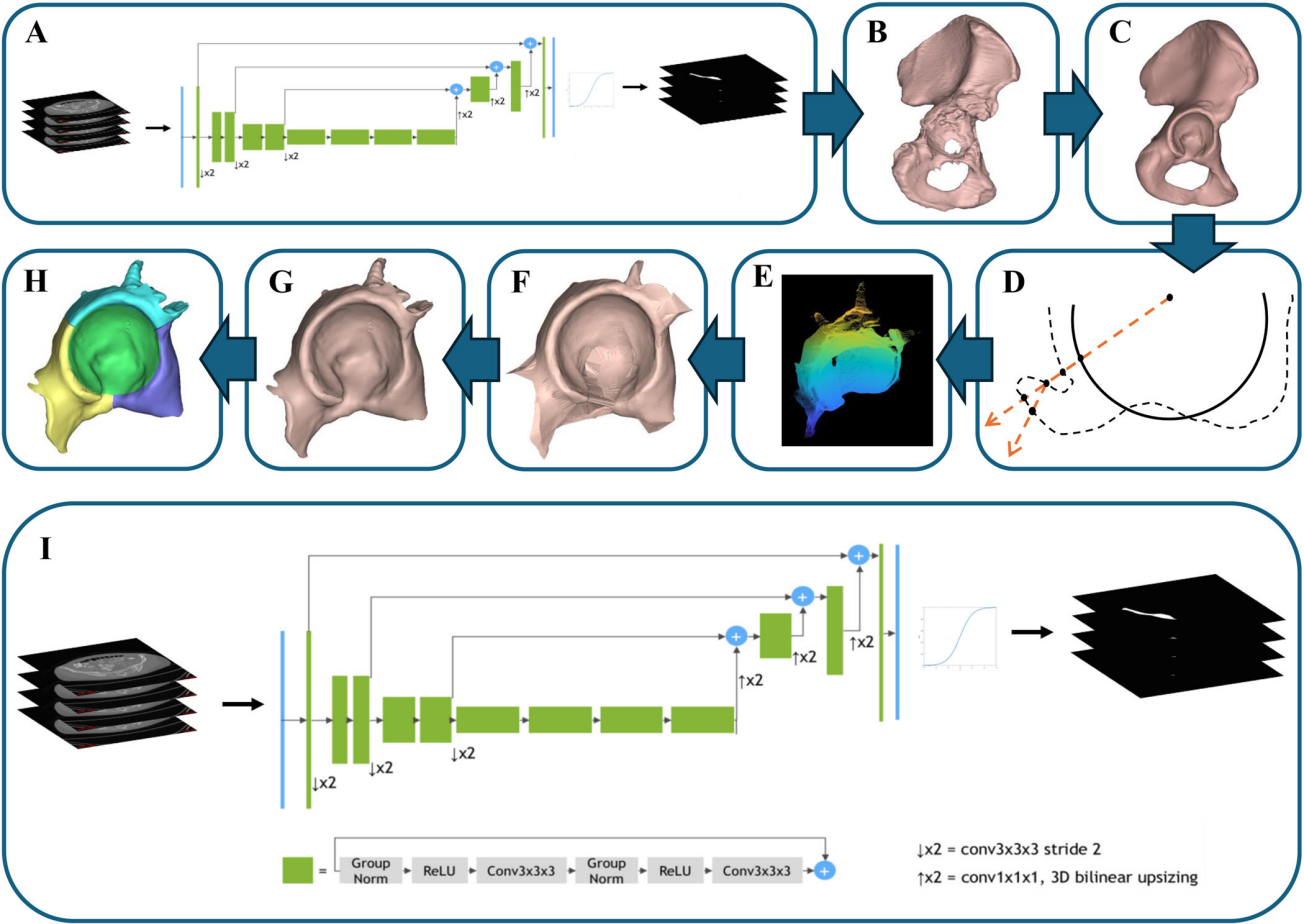
Automated modeling pipeline flow diagram including (A) automated neural network segmentation from preoperative computed tomography (CT) images (B) generation of 3D model of remaining pelvic bone from neural network segmentation (C) native, defect‐free pelvis generated from statistical shape model (D) ray casting (E) point cloud of missing acetabular defect bone generated from ray casting (F) alphashape of acetabular defect (G) acetabular defect model after mesh Boolean operations (H) acetabular defect model after regional definition, and (I) SegResNet architecture used in neural network segmentation. SegResNet architcture figure reprinted with permission from Spring Nature publishing.

An automated computational modeling framework employing artificial neural networks, statistical shape modeling (SSM) and a ray‐casting approach was developed to digitize anatomy of the pelvis and acetabular bone defect from the CT scans and applied retrospectively to all included patients (Figure 1A–H). An artificial neural network was first adopted to automatically reconstruct 3D pelvic anatomy using preoperative CT images. Utilizing the Pytorch machine learning library [13], multiple neural network architectures were tested and evaluated, with a modified 3D SegResNet architecture [14] being selected for full hyperparameter optimization (Figure 1I). To reduce memory usage, CT images were automatically cropped to include only the affected hemipelvis. Image stacks greater than 384 slices in height were downsampled to 384 slices, roughly equivalent to a slice thickness of 0.6 mm.

The input to the artificial neural network was a 3D array consisting of the Hounsfield units of each voxel in the cropped volume, while the output was a binary segmentation mask of the same volume. Image datasets were first randomly split into three groups of 20 images. Each group was then used once as the inference data set in a 5 k‐fold cross validation with a 32 training, eight validation and 20 inference data spilt. This resulted in 15 trained networks, one for each fold in the three groups, with the best performing fold being selected as the final network for each group. This resulted in three trained artificial neural networks and ensured no inference subjects were used to train or validate the neural network applied to them, while still employing all subjects. A postprocessing algorithm was then applied to compare the segmentations masks to the preoperative images and generate an STL of each pathological pelvis inclusive of acetabular defect.

To reconstruct the acetabular defect in each patient, a predictive reconstruction of the patient's native, defect‐free pelvis was required. To achieve this, a SSM of a healthy right hemipelvis was developed using the GIAS2 software package [15] and 57 healthy pelvis CT image datasets from the Cancer Image Archive [16] (22 females, 35 males, age: 49.0 ± 14.8 years). Only the right sided hemipelvises from these images were used in SSM development, and the first 20 modes were used to reconstruct the native pelvis from the pathological pelvis. The use of SSMs in this manner has been shown to reproduce defect‐free bony anatomy of the acetabulum with an RSME of 0.83 ± 0.17 mm [17]. The 3D reconstructions of the pathological pelvises, inclusive of acetabular defects, were input into the SSM, resulting in a defect‐free native pelvic anatomy estimation. To prevent influence from pathological bone on the SSM‐generated estimation of native pelvic anatomy, a MATLAB (Mathworks, US) sphere fitting algorithm was then used on 19 automatically selected points on the native acetabular rim to calculate the native hip joint centre, acetabular radius, acetabular plane and acetabular polar axis. A sphere of radius 2.5 times the acetabular radius was generated at the hip joint centre and a mesh Boolean operation used to remove any pathological bone anatomy inside this sphere volume from the pathological pelvis model. The value of 2.5 times acetabular radius was used as this was the radius required on the largest defect in the data set. This new model was then refitted to the SSM to derive a more accurate native pelvis estimation.

Once 3D reconstructions of the pathological pelvis and native defect‐free pelvis were obtained, a custom ray casting algorithm was used to calculate the difference between the two geometries and subsequently generate an initial model of the acetabular defect. A spherical coordinate system was defined, and an angular spread of ray vectors calculated. Each ray was cast into both the native and pathological pelvis models and the distance to the intersections of the rays in each model recorded. As multiple entry and exit intersections are possible, a maximum limit of four intersections was set. Intersections beyond 2.5 times the acetabular radius were also excluded. From these conditions a series of logic rules were defined to simulate scenarios and record relevant points in 3D space (Supporting Information: Table S1). The points were then segmented into clusters. Clusters with less than 200 points were discarded. The remaining points were converted to a STL, and any holes closed using a mesh reconstruction. Lastly, two mesh Boolean operations were performed to remove errors created in the STL conversion and mesh reconstruction steps. A subtractive Boolean with the pathological pelvis model improved accuracy at the defect/pathological pelvis boundary, while an intersection Boolean with the native pelvis model improved accuracy of the acetabular articular surface.

### 2.3 Acetabular Defect Analysis

**Figure 2:**
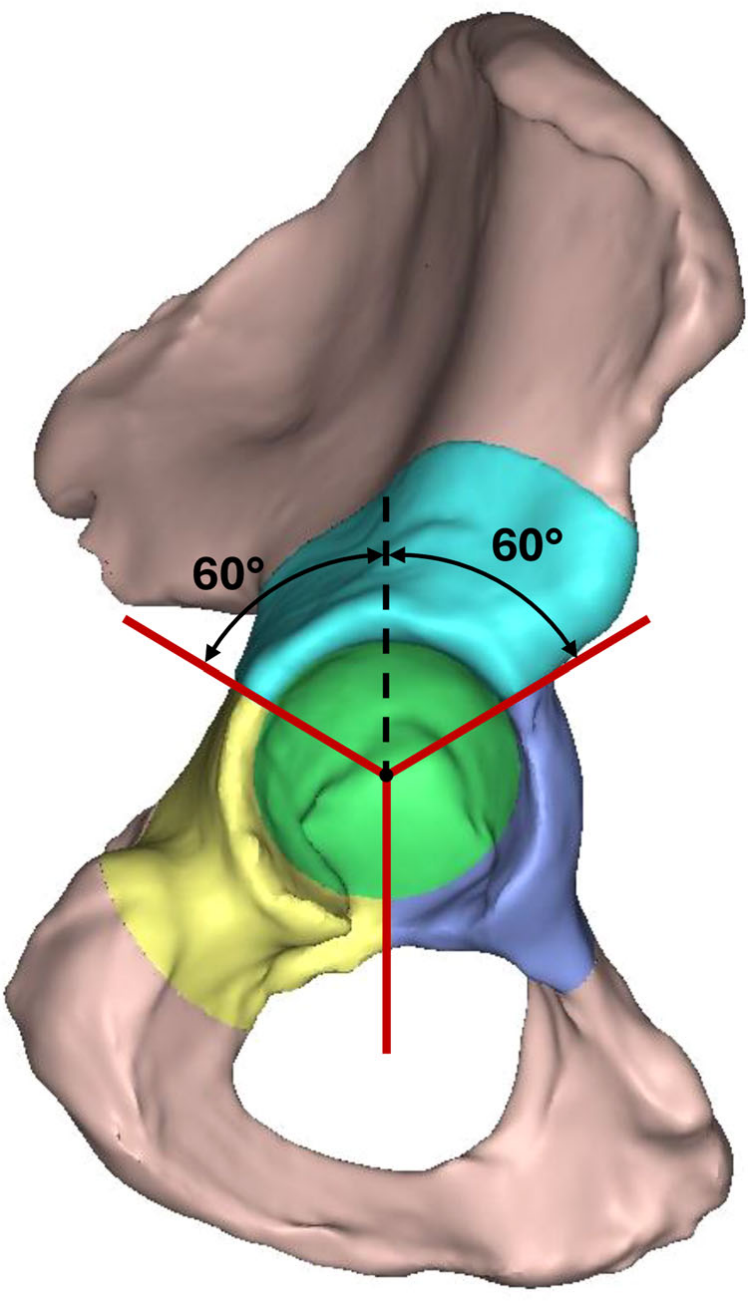
Acetabular regions defined for the pelvis including superior region (teal), posterior region (yellow), anterior region (purple), and medial wall region (green). Dotted line represents vertical axis of defined acetabular coordinate system.

The reconstructed acetabular defect models were divided into four anatomical regions and the absolute volume, relative volume and defect depth in each region calculated. An acetabular coordinate system described by Grace et al. [18] was used to define the superior, posterior, anterior and medial wall regions [18] (Figure 2). These regions extended 2.2 times the acetabular radius from the hip joint centre for the superior region and 1.9 times the acetabular radius for the anterior and posterior regions. The medial wall region was defined as a cylindrical volume aligned with the acetabular polar axis with a radius of 0.86 the acetabular radius [19]. Relative defect volumes were calculated by comparing the regional defect volume with the regional native volume while defect depth was calculated as the distance from the furthest valid intersection in the patholgoical pelvis model to the first intersection of the native pelvis model along each casted ray.

Manually derived acetabular defect models were compared with automatically generated acetabular defect models. To achieve this, manually segmented pathological pelvis models inclusive of defects were first produced, followed by the creation of a sphere 2.5 times the acetabular radius at the hip joint centre. These models were then used in a mesh Boolean operation to remove the pathological defect region inside the sphere from the pathological pelvis model. This modified pathological pelvis model was fitted to the SSM, and a native pelvis estimation generated. The native pelvis was then imported to Mimics for comparison with the original manually segmented pathological pelvis. A mesh Boolean operation removing the pathological pelvis from the native pelvis was applied, followed by manual segmentation to derive a reconstruction of the acetabular defect. This manually generated acetabular defect model was then split into four regions with absolute volumes, relative volumes and defect depths of each region calculated for comparison with the automatic‐generated acetabular defect models.

The geometric accuracy of the automatically generated acetabular defect models was assessed using volumetric dice coefficient, volume mean absolute error (MAE), volume mean absolute relative error (MARE) and Hausdorff distance. Volumetric dice coefficient was calculated by directly comparing the 3D binary segmentation masks of the automatically generated acetabular defect models with those of the manually generated acetabular defect models. Volume and Hausdorff distance were calculated using the PyMeshLab [20] package. The pathological pelvis models were stratified by defect classification and the total and regional relative defect volumes (RDV), absolute defect volumes (ADV) and defect depths (DD) of each corresponding acetabular defect model were calculated within each classification group.

### 2.4 Statistical Analysis

A Shapiro–Wilk test was performed to determine data normality, and Levene's test was performed to assess equality of variances within defect classification groups. A Kruskal–Wallis test with post hoc comparisons was then used to evaluate statistical difference between defect classification group means with significance set to *p* < 0.05. 2A defects were excluded from statistical comparisons due to small sample size (*n* = 2). Standard deviation was used as a measure of data dispersion.

## 3 Results

### 3.1 Acetabular Defect Reconstruction Accuracy

The automatically reconstructed acetabular defect models had a mean volumetric dice coefficient of 0.827, a mean absolute error of 11.0 cm^3^, a mean absolute relative error of 16.4%, and a mean Hausdorff distance of 2.8 mm (Table 2). The modeling framework took approximately 15 min to reconstruct a patient's acetabular defect from preoperative CT images. Neural network segmentation of the complete pathological hemipelvises of rTHA patients was performed with a mean dice coefficient of 0.948 (Supporting Information: Table S2), and took approximately 3 s to reconstruct each hemipelvis.

**Table 2:** Volumetric dice coefficients, MAREs, MAEs and Hausdorff distances for each defect classification group and across all defect classifications (overall).

	Dice coefficient	Volume MAE (cm^3^)	Volume MARE (%)	Hausdorff distance (mm)
2A	0.774	7.95	22.2	2.51
2B	0.754	8.65	18.6	3.98
2C	0.821	10.1	18.1	2.82
3A	0.887	8.74	12.9	2.19
3B	0.870	15.9	15.4	2.39
Overall	0.827	11.0	16.4	2.81

Abbreviations: MAE, mean absolute error; MARE, mean absolute relative error.

**Figure 3:**
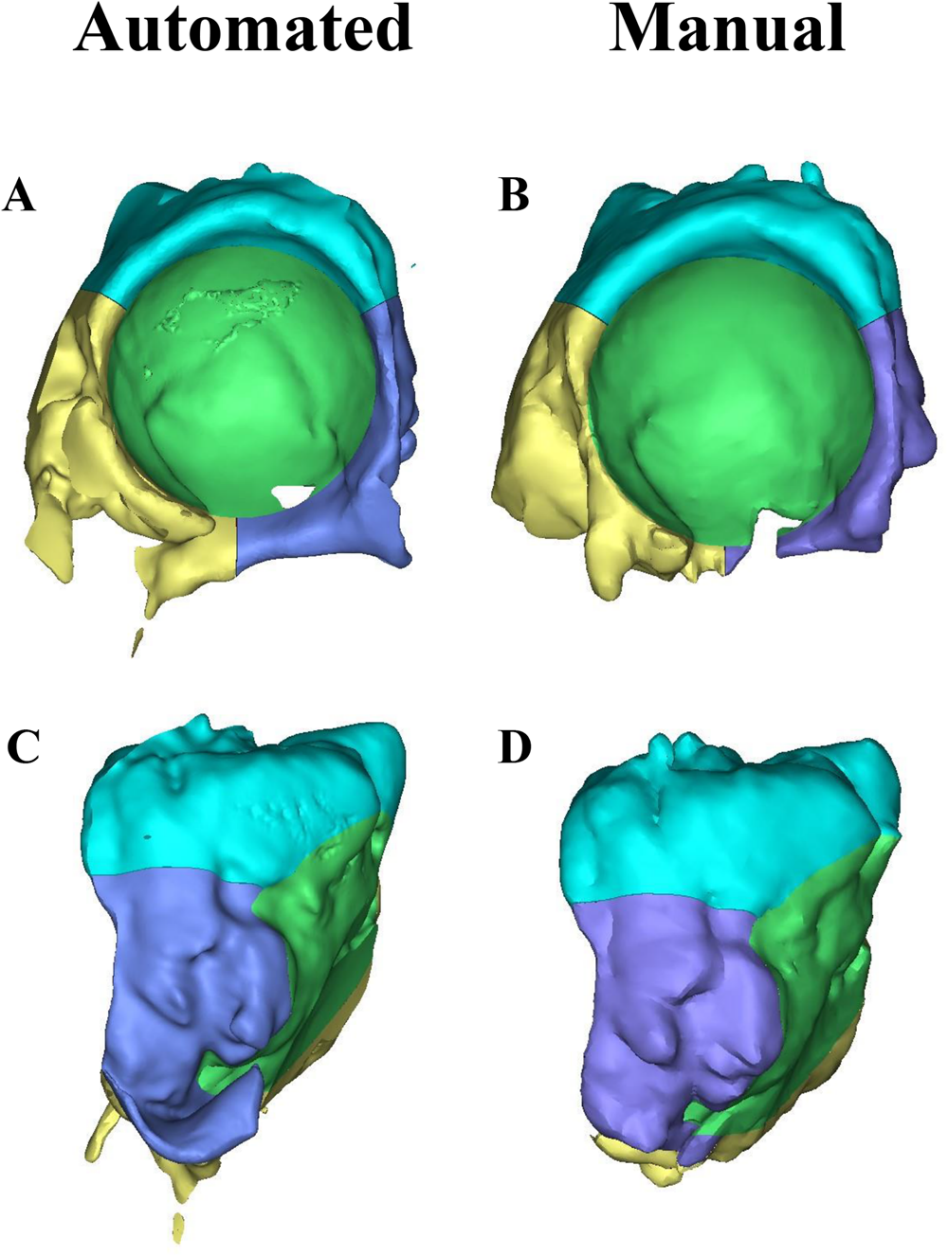
Automated acetabular defect model generated by modeling pipeline and corresponding manually developed models including (A) frontal view of automatically generated acetabular defect model (B) frontal view of manually generated defect model (C) side view of automatically generated acetabular defect model, and (D) side view of manually generated defect model.

Reconstructions of larger defects (Paprosky 3 A and 3B) had higher dice coefficients than smaller defects (Paprosky 2A–2C) (Figure 3). For example, the mean dice coefficient of 3B defects was 0.849, compared to 0.754 for 2B defects (median difference: 0.098, 95% confidence interval [CI]: [0.018–0.156], *p* = 0.005). 3A defects had the lowest mean MARE of 12.9% (MAE: 8.74 cm^3^), while the highest mean MARE (excluding 2A defects) was 18.6% in 2B defects (MAE: 8.65 cm^3^) (median difference: 3.8%, 95% CI: [−11.5 to 7.3], *p* > 0.05). The highest mean Hausdorff distance was 4.0 mm for 2B defects, compared with a lowest of 2.2 mm for 3A defects (median difference: 1.8 mm, 95% CI: [0.4–3.0], *p* = 0.015).

### 3.2 Defect Morphology

**Figure 4:**
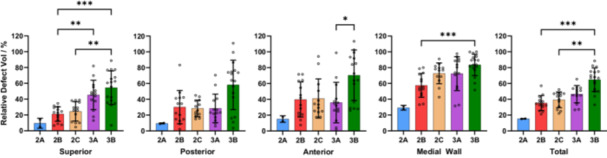
Mean regional and overall relative defect volumes of each Paprosky defect classification group. Whiskers represent one standard deviation.

**Figure 5:**
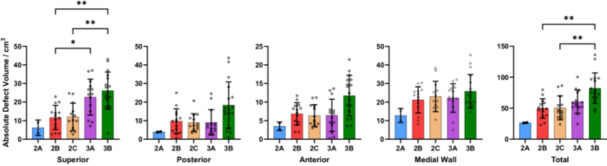
Mean regional and overall absolute defect volumes of each Paprosky defect classification group. Whiskers represent one standard deviation.

**Figure 6:**
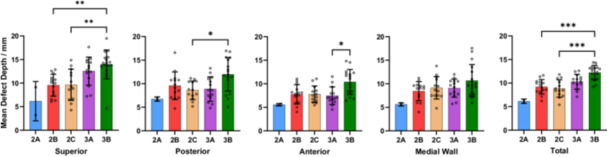
Mean regional and overall defect depths of each Paprosky defect classification group. Whiskers represent one standard deviation.

When considering the entire acetabular defect geometry, increased defect severity was associated with increases in mean RDV (range 2A–3B: 15.3%–64.7%, *p* < 0.001), ADV (range 2A–3B: 26.5–82.2 cm^3^, *p* < 0.001) and DD (range 2A–3B: 6.1–12.2 mm, *p* < 0.001) (Table 3). When comparing mean RDV, ADV and DD between defect classifications, significant differences were only found between 3B defects and 2B–2C defects (*p* < 0.05). (Figures 4, 5, 6) Significant differences in mean RDV values were observed between 3B and 2B defects (median difference: 30.4%, 95% CI: [19.5–40.5], *p* < 0.001) and between 3B and 2C defects (median difference: 23.5%, 95% CI: [15.9–35.8], *p* < 0.001). Significant differences in mean ADV values were also observed between 3B and 2B defects (median difference: 26.7 cm^3^, 95% CI: [17.2–46.7], *p* < 0.001) and 3B and 2C defects (median difference: 31.0 cm^3^, 95% CI: [13.8–46.4], *p* < 0.001). Significant differences in mean DD values were found between 3B and 2B defects (median difference: 3.3 mm, 95% CI: [1.9–4.3], *p* < 0.001) and 3B and 2C defects (median difference: 4.2 mm, 95% CI: [2.0–4.8], *p* < 0.001).

**Table 3:** Mean absolute defect volume (cm^3^), mean defect depth (mm) and defect volume as a percentage of total defect size (%) for each acetabular region and Paprosky defect classification group (2A–3B).

	Total			Superior			Posterior			Anterior			Medial wall		
	%	cm^3^	mm	%	cm^3^	mm	%	cm^3^	mm	%	cm^3^	mm	%	cm^3^	mm
2A	15.3	26.5	6.2	9.5	6.17	6.2	9.54	4.0	6.7	15.2	3.5	5.6	29.3	12.8	5.6
2B	35.2	49.6	9.22	21.1	11.7	9.6	30.8	9.7	9.6	39.9	6.8	7.8	57.6	21.2	8.4
2C	39.4	50.5	8.8	24.5	12.1	9.7	28.5	9.0	8.6	41.1	6.4	7.8	72.8	23.0	9.1
3A	46.5	60.4	10.3	45.3	22.7	12.6	28.5	9.1	8.9	35.6	6.4	7.5	72.5	22.2	9.1
3B	64.7	82.2	12.2	54.5	26.1	13.9	58.3	18.4	12.0	70.5	11.7	10.4	83.5	25.8	10.7
Combined	46.4	60.8	10.1	36.6	18.2	11.4	36.7	11.7	9.79	47.1	7.92	8.4	70.7	22.9	9.3

Regional measurements of mean RDV, ADV, and DD followed the same increasing trend with greater defect severity; however, comparisons of regional quantitative measures between defect groups resulted in few statistically significant differences. Apart from the comparison of mean RDV and ADV between 3A and 2B defects in the superior region, and mean RDV and DD between 3B and 3A defects, the only significant differences were found between 3B and smaller defects (2B–2C). Significant differences (*p* < 0.001) were found in superior RDV between 3B and 2B defects (median difference: 37.4%, 95% CI: [20.1–47.8]), and in medial wall RDV between 3B and 2B defects (median difference: 28.6%, 95% CI: [15.4–37.2]).

## 4 Discussion

The objective of this study was to develop a pipeline to automatically generate models of rTHA acetabular defects from preoperative CT scans, and use this to assess defect volumes and depths across Paprosky defect classifications. Comparison of the automatically generated acetabular defect models relative to manually derived defect models resulted in a mean dice coefficient of 0.827 and a mean volume MARE of 16.4%, indicating a similar level of accuracy to other automated pathological bone reconstructions [21, 22]. The segmentation results also indicated that an artificial neural network using supervised training can produce accurate segmentations from clinical CT datasets affected by metal artifact with high levels of repeatability. Over the time period of data collection (2010–2023), changes to CT characteristics and improvements to metal artifact reduction techniques may have occurred; nonetheless, the neural network architecture employed was robust to this data heterogeneity and no significant differences in acetabular defect reconstruction accuracy were observed in relation to year of preoperative CT imaging.

The results demonstrate that our automated reconstructions of large acetabular defects had higher dice coefficients than those of small defects. An explanation for this may be that both the automated and manual defect reconstruction methods use a SSM estimation of native pelvis anatomy. When defects are severe and large volumes of bone are missing from the pathological acetabulum, both methods will share a similar SSM generated estimation of these missing volumes, resulting in high dice coefficients when comparing the two. The highest dice coefficients and MAREs therefore occurred when there was very little or no acetabular bone remaining. Smaller defect volumes and few holes would be expected in the acetabulum of a 2A or 2B defect, which may explain why these defects had lower accuracies than 3A and 3B defects. Additionally, small topological differences between the manual and neural network‐generated pathological pelvis models would have a larger negative effect on the accuracy of smaller 2A–2C defects than large 3A or 3B defects. The higher MAREs of the smaller defect classifications could also be explained by the smaller absolute sizes of those defects. Improving the accuracy of the initial hemipelvis segmentation via improved metal artifact reduction techniques, or with the use of a larger image data set, may ultimately improve the accuracy of small acetabular defect reconstruction.

Relative defect volumes, absolute defect volumes and defect depth generally increased with defect size. The medial wall region showed the highest mean relative bone losses of all regions (2A: 29.3%–3B: 83.5%) while the smallest mean relative volume losses were in the superior region (2A: 9.5%–3B: 54.5%); however, this represents the difference in absolute native region volume between the two since the superior region had the highest absolute mean bone loss volume (3B: 26.1 cm^3^). Mean relative and absolute defect volumes of 2A and 2B defects were highest in the medial wall region with 29.3% and 12.8 cm^3^, and 57.6% and 21.2 cm^3^, respectively. The 2B values were higher than most mean 2A–3A defect volumes in all other regions, which may indicate that the medial wall loses the most bone volume in primary THA due to reaming in the medial direction.

**Figure 7:**
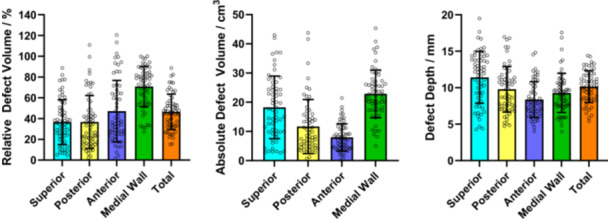
Relative defect volumes, absolute defect volumes and effect depths of all subjects in each acetabular region. Columns represent mean value and whiskers represent one standard deviation.

The results show that all rTHA subjects incurred significant multi‐directional bone loss. The smallest total RDV was 15.2% and every subject had a mean defect depth of at least 4 mm in all regions, a finding that was consistent with reaming depth studies of primary THA procedures [23] (Figure 7). In almost all cases, mean defect depths were highest in the superior region (2A: 6.2–3B: 14.0 mm), although the mean defect depth in the superior region was lower than the Paprosky threshold of superior bone loss in 3B defects, which stipulates acetabular cup superior migration of over 30 mm. There are several possible reasons for this disparity. Firstly, defect depths calculated within this study were assessed in a 3D manner as a mean across the entire defect region using the estimated native acetabulum as a reference, and maximum depths may therefore be significantly higher than those of the mean. This contrasts with the Paprosky classification which was designed for assessment of planar radiographs in which the maximum depth may be taken as defect depth. Secondly, misdiagnosis may be a factor with smaller defects being classified as a 3B defect. Lastly, estimation of bone loss relative to the native acetabulum may be difficult and prone to overestimation due to previous index primary arthroplasty and in many cases additional previous revision arthroplasties.

There were large variations in defect volumes and depths within each Paprosky defect classification. In all acetabular regions except the medial wall, the variation in RDV, ADV, and DD generally increased with defect severity (Figures 4, 5, 6). The greatest variability in RDV was found in the 3B anterior region (standard deviation: 30.9%) while Paprosky 2C defects showed the smallest variability across all regions (mean standard deviation: 13.6%,), when excluding 2A defects due to sample size. When considering variability of volume and depth, there was significant overlap between defect classifications 2B, 2C, and 3A, especially in the posterior, anterior and medial wall regions. This finding, which supports the study hypothesis, may indicate difficulty in diagnosing these defects using standard clinical imaging, which has potential implications for preoperative planning. It may also indicate that the Paprosky classification may be overly dependent on the presence of specific anatomical landmarks such as the acetabular teardrop and Kohler's line, as well as the shape of the defect, which volume and depth measures do not consider.

There were few statistically significant differences in ADV, RDV, and DD between Paprosky classifications in all regions due to high variability of these values within each classification group. Multiple significant differences in volume and depth were found between 3B and 2B defects due to the large differences in defect severity between these groups. However, significant differences between 2B and 3A defects were only observed in superior ADV (*p* = 0.05) and superior RDV (*p* = 0.009), while significant differences between 3A and 3B classifications were only observed in anterior RDV (*p* = 0.04) and anterior DD (*p* = 0.02), indicating that the quantitative measures investigated in this study cannot be used to distinguish between consecutive Paprosky defect severities. This may lead to the conclusion that the acetabular regions defined in this study and previous studies may not be suitable for comparison with the Paprosky classification system, though we do not believe this to be the case. The defined regions generally align with the four regional domains of the Paprosky classification, as the presence or absence of bone in these regions is believed to be integral to achieving initial implant stability, a known surrogate of longevity [24, 25]. The lack of correlation between the Paprosky classifications and ADV, RDV, and DD may reflect limitations in viewing or quantifying bone loss adjacent to metallic implants using medical imaging. Particularly as the Paprosky system was proposed for radiographs before the widespread use of CT. Alternative classification systems have been proposed [7], including the AAOS, Gross and Gustilo classifications, but their association with quantifiable defect volume and depth data has not yet been evaluated.

The results of this study are in reasonable agreement with previously published research. Grace et al. investigated intra‐ and inter‐operator repeatability of calculated lesion volume in a cadaveric specimen using a semi‐automated method. They recorded intra and inter‐observer variations of volume MAREs in a unilaterally implanted specimen of 1.2% and 5.7%, respectively [18] and intra and inter‐observer variations of volume MAREs in a bilaterally implanted specimen were 2.2% and 7.1%, respectively. These results are an improvement on the 16.4% volume MARE in the present study; however, their method only assessed one cadaveric specimen (when unilaterally and bilaterally implanted) and the approach was not automated and also involved manual image segmentation. Schierjott et al. used manual image segmentation and SSMs to quantitatively analyse 50 acetabular defects, finding that relative bone loss was highest in the medial wall region [26]. While direct comparison with the present study of defect classification group means of ADV and RDV was not possible as the defect classifications were not reported, there was general agreement between the mean absolute and relative defect volumes of both studies (Table 4).

**Table 4:** Mean total and regional defect volume for subjects in the present study and those from Schierjott et al. [26].

	ADV (cm^3^)		RDV (%)	
	Schierjott et al.	Present study	Schierjott et al.	Present study
Superior	21.2 ± 11.2	18.2 ± 10.6	47.6 ± 26.3	34.6 ± 21.0
Posterior	13.8 ± 8.4	11.7 ± 9.2	41.4 ± 24.9	32.8 ± 21.6
Anterior	7.5 ± 3.6	7.9 ± 4.6	42.6 ± 20.5	42.7 ± 26.4
Medial wall	14.4 ± 5.7	22.9 ± 8.1	69.2 ± 24.8	69.1 ± 19.5
Total	56.8 ± 21.6	60.8 ± 24.3	48.7 ± 18.6	43.7 ± 15.0

Abbreviations: ADV, absolute defect volumes; RDV, relative defect volumes.

The modeling pipeline proposed in this study automatically reconstructed acetabular defect morphology and volume rapidly and with high accuracy. Creation of the defect models took approximately 15 min per patient when using 8 Intel Xeon Gold 6326 (2.9 GHz) processors and 1 Nvidia A100 GPU (80GB). The most time‐consuming step was ray casting, though processing time could be further reduced by deploying additional processors. Manual segmentation of acetabular defect cases can be time consuming, even when using semi‐automated tools, taking between 2 h and 2 days depending on operator experience and scan quality. This study provides a framework to rapidly generate estimations of region‐specific defect anatomy and volume, as well as other anatomically relevant parameters such as HJC and acetabular inclination and anteversion, which may be useful in surgical planning. The tools may be employed in the design of custom implants, although further consideration would need to be applied to implant insertion trajectories, screw trajectories and bone quality, which would all play a part in custom implant longevity.

There are several limitations to this study. First, CT images heavily affected by metal artifact were not included in the study, and the decision to exclude certain images was subjective and based on capacity to sufficiently delineate the boundary between acetabular trabecular bone and surrounding tissue. Most modern imaging systems are equipped with artifact reduction protocols, which would ultimately assist in achieving higher acetabular defect reconstruction accuracy using the proposed modeling tools. Second, the native pelvis models derived from the SSM may contain topological errors when compared to the actual native anatomy, especially in cases of abnormal native anatomy such as femoroacetabular impingement or hip dysplasia. However, the reconstruction pipeline aims to estimate the morphology and volume of missing healthy bone to aid rTHA outcomes, and recreation of abnormal non‐defect related anatomy would not typically be an objective of rTHA procedures. Third, our modeling framework detected bone loss due to screw holes from previous procedures, the volume of which would contribute to the total volume of missing bone calculated. Despite this, bone void due to orthopaedic screws is likely to be small and not influence the major findings of between‐defect grade differences in defect morphology. Lastly, this study trained and utilized three artificial neural networks to utilize all suitable CT images. Differences in hemipelvis segmentation and defect reconstruction accuracies were small, indicating that the pipeline architecture is robust to changes in training data. It is reasonable to expect better performance from a single neural network trained on a larger data set.

A fast and automated computational pipeline was developed to calculate acetabular defect morphology and volume in rTHA patient CT data sets. High reconstruction accuracy and reproducibility was demonstrated across different image modalities and all defect classification grades. Correlation between ADV, RDV, and DD and Paprosky classification was poor, and distinguishing between defect classifications based on regional defect volumes was not possible. Additionally, the volume of bone loss in 2A defects was found to be considerable, likely due to bone loss in a primary THA procedure. The findings of this study may be useful in surgical planning and guiding reconstruction strategies for rTHA in the presence large acetabular defects.

## Supporting information


**Table S1:** Logic rules used to determine defect and native pelvis points during ray‐casting procedure. **Table S2:** Segmentation and acetabular defect modelling accuracy of the three trained neural networks. **Figure S1:** Representative CT image of excessive metal artifact leading to exclusion of patient from dataset.
