# Supplementary material for: Automated Acetabular Defect Reconstruction and Analysis for Revision Total Hip Arthroplasty: A Computational Modeling Study
Source: J Orthop Res. 2025 May 2;43(7):1315–24. doi: 10.1002/jor.26086 (PMC12159582; doi:10.1002/jor.26086)
Supplement: Supplementary file 1 — Table S1: Logic rules used to determine defect and native pelvis points during ray‐casting procedure. Table S2: Segmentation and acetabular defect modelling accuracy of the three trained neural networks. Figure S1: Representative CT image of excessive metal artifact leading to exclusion of patient from dataset. [file JOR-43-1315-s001.docx]

**Supplementary Material**

**Table S1:** Logic rules used to determine defect and native pelvis points during ray-casting procedure.

|  | **Ray Casting Logic Rules** |
| --- | --- |
| 1. | 1^st^ defect intersection is further than 1^st^ native intersection – defect present. Record 1^st^ native and 1^st^ defect intersection point. |
| 2. | 1^st^ defect intersection is further than 2^nd^ native intersection – defect present. Record 1^st^ native and 1^st^ defect intersection point. This scenario can occur due to surface inaccuracies between the native and defect models or abnormal bone remodelling in the presence of an implant. |
| 3. | No defect intersection within 2.5 acetabular radii of HJC and 2 native intersections present – defect present, no native bone remaining. Record 1^st^ and 2^nd^ native intersection point. |
| 4. | 1^st^ defect intersection is closer than 1^st^ native intersection – no defect. Do not record any intersection points. |
| 5. | More defect intersections present than native intersections – defect present. This scenario indicates the presence of obscured surfaces within the defect surface. Record 1^st^ native intersection and 1^st^ defect intersections. Cast ray from 2^nd^ and 3^rd^ defect intersections to differentiate between obscured defect surface and surface deviations on outer pelvis. Rays from obscured defect surface should intersect with defect pelvis, if so, record 2^nd^ and 3^rd^ defect intersection points if intersection from these points occur. |
| 6. | More native intersections present than defect intersections – defect present. This scenario occurs when native rays pass through the acetabular rim and then re-enter the pelvis and when no acetabular rim is present on the defect pelvis. Record 1^st^ and 2^nd^ native intersections and treat 1^st^ defect and 3^rd^ native intersection along scenarios 1-5. |

**Table S2:** Segmentation and acetabular defect modelling accuracy of the three trained neural networks.

|  | **Neural Network Segmentation Accuracy** | | | | | **Acetabular Defect Model Accuracy** |
| --- | --- | --- | --- | --- | --- | --- |
|  | **Fold 1** | **Fold 2** | **Fold 3** | **Fold 4** | **Fold 5** |  |
| **Group 1** | 0.947 | 0.948 | 0.949 | 0.949 | 0.949 | 0.797 |
| **Group 2** | 0.947 | 0.949 | 0.949 | 0.949 | 0.948 | 0.866 |
| **Group 3** | 0.945 | 0.942 | 0.943 | 0.941 | 0.945 | 0.819 |

**
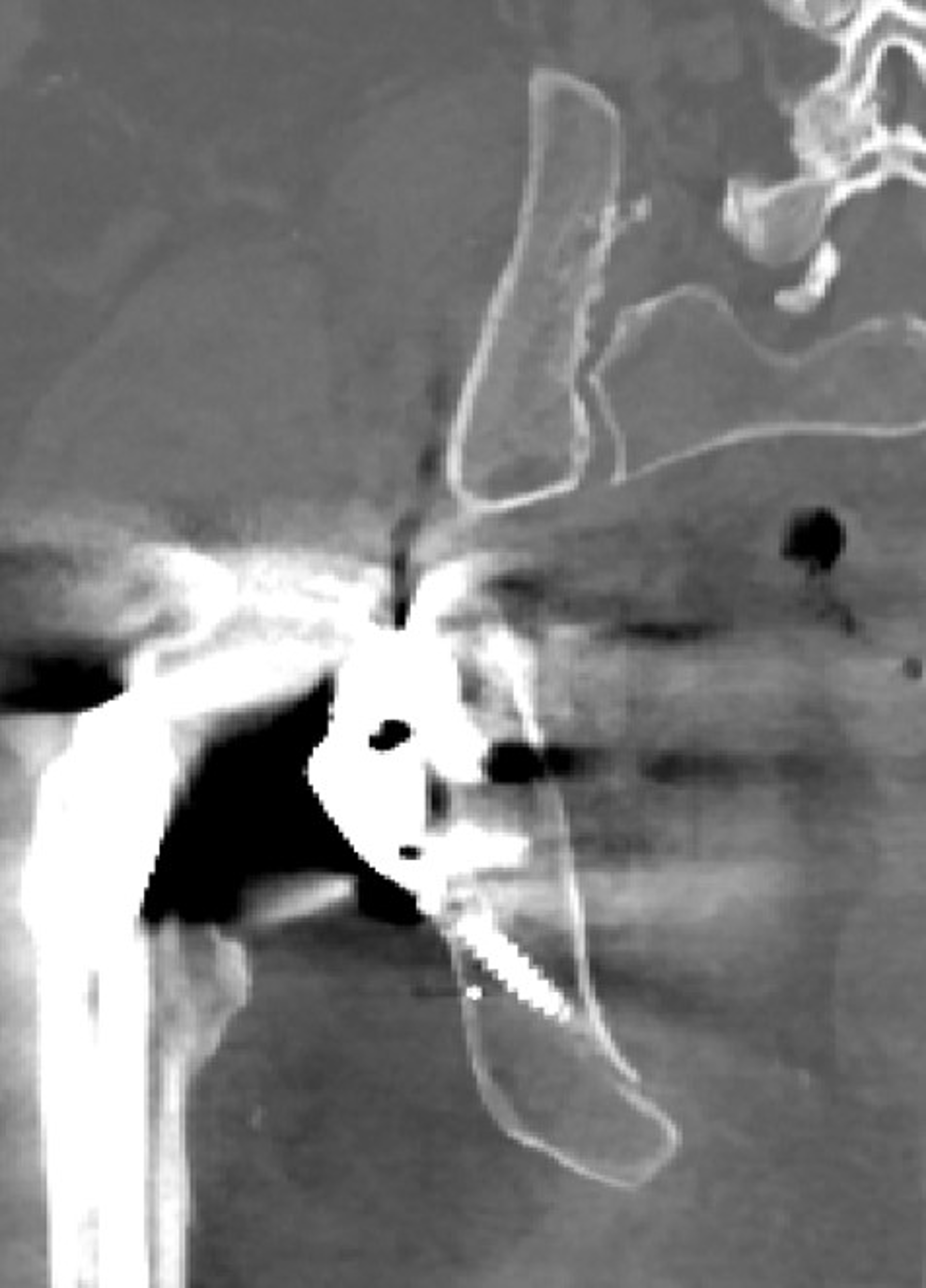
**

**Figure S1:** Representative CT image of excessive metal artifact leading to exclusion of patient from dataset.
